# Magnetic Nanowires for Nanobarcoding and Beyond

**DOI:** 10.3390/s21134573

**Published:** 2021-07-03

**Authors:** Mohammad Reza Zamani Kouhpanji, Bethanie J. H. Stadler

**Affiliations:** 1 Department of Electrical and Computer Engineering, University of Minnesota, Minneapolis, MN 55455, USA; zaman022@umn.edu; 2 Department of Biomedical Engineering, University of Minnesota, Minneapolis, MN 55455, USA

**Keywords:** magnetic nanowires, nanobarcodes, encoding, sensing and decoding

## Abstract

Multifunctional magnetic nanowires (MNWs) have been studied intensively over the last decades, in diverse applications. Numerous MNW-based systems have been introduced, initially for fundamental studies and later for sensing applications such as biolabeling and nanobarcoding. Remote sensing of MNWs for authentication and/or anti-counterfeiting is not only limited to engineering their properties, but also requires reliable sensing and decoding platforms. We review the latest progress in designing MNWs that have been, and are being, introduced as nanobarcodes, along with the pros and cons of the proposed sensing and decoding methods. Based on our review, we determine fundamental challenges and suggest future directions for research that will unleash the full potential of MNWs for nanobarcoding applications.

## 1. Introduction

Initially, barcodes were invented for the authentication of products in anti-counterfeiting, which is of the foremost importance due to the continuous growth of non-transparent trading [1,2,3,4]. Nanostructured materials are the backbone in barcoding applications, because their similar appearance hides them from the naked eye, while their physical and chemical properties are significantly different and are suitable for authentication [5,6]. As a result, the unmet demands for miniaturized barcodes led to the emergence of nanobarcodes, such as magnetic nanoparticles [7,8,9], magneto-optic nanoparticles [10,11], and photonic nanoparticles [12,13,14], in diverse applications, including nanomedicine and cell biology [15,16,17,18], as well as computing and cryptography [19,20,21]. Changing the composition and size of nanomaterials is probably the most convenient approach to generate numerous nanobarcodes with distinct codes [22]. However, generating nanobarcodes with unique codes does not necessarily guarantee that reliable sensing and decoding is also possible, especially when there is more than one nanobarcode at the scanner. This restriction obligates defining the essential merits for nanobarcodes and designing nanobarcodes that meet these merits [5,6].

**Figure 1:**
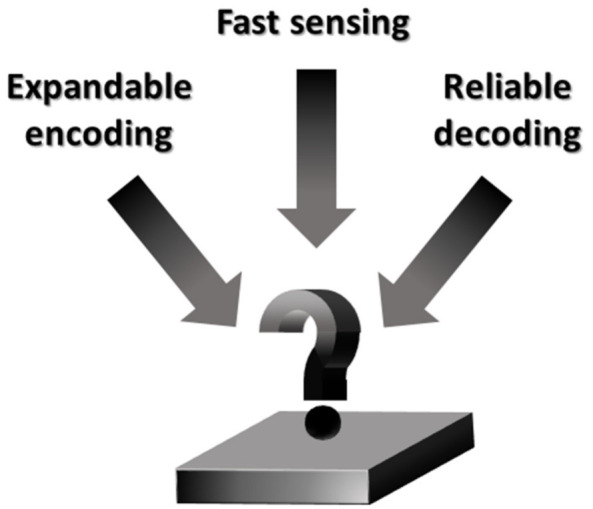
A flowchart rendering the essential merits for nanobarcodes.

In the big picture, there are the following three essential merits for the ideal nanobarcode: (1) expandable encoding, (2) secure sensing, and (3) reliable decoding, as shown in Figure 1. Simply, the codes are physical properties with high flexibility that can be easily tailored and measured. For many applications, the sensing must be done by non-destructive measurement techniques with high repeatability [23] that can be readily translated to the portable devices, suitable for daily applications. Therefore, the first two essential merits are strongly correlated, and they can be tackled by choosing nanomaterials/nanostructures with special properties, which can be readily engineered and measured. The first two merits may discard several proposed nanomaterials/nanostructures for nanobarcoding, but there is still a vast number of nanomaterials/nanostructures that meet these two merits and are deemed promising. To specialize this review, here, we only focus on one-dimensional magnetic nanoparticles, also known as magnetic nanowires (MNWs), with ferromagnetic properties, to deeply discuss their recent progress, particularly in nanobarcoding applications and how they potentially can transform the future of this field. 

## 2. Why Magnetic Nanowires for Nanobarcodes?

Recently, MNWs became the center of the research in nanobarcoding because of the revealed potential for making the next generation of nanobarcodes and/or biolabels [23,24,25,26], driven by the fact that the MNWs can be remotely and selectively detected [27,28,29]. Moreover, the MNWs are dominantly fabricated using electrodeposition techniques that are cheap, fast, and scalable for mass production [30,31,32,33]. More importantly, their magnetic response can be readily extracted from the background signals, leading to a high signal-to-noise ratio–suitable for miniaturizing the barcodes size [7]; this is because the majority of materials are diamagnetic or paramagnetic, which do not produce magnetic signals, such as irreversible switching, which is exclusively a property of ferromagnetic materials [34,35]. Thus, as opposed to optical- or radio-frequency barcodes, magnetic signals are not contaminated by the background noise [36,37,38,39]. Aside from these advanced benefits of MNWs for nanobarcoding, they also meet the aforementioned merits of expandable encoding, fast sensing, and reliable decoding, which we discuss in detail in the following sections. First, due to the strong correlation between the encoding merit and the sensing merit, we discuss these merits together. We next discuss the current state-of-the-art for the reliable decoding of multiple MNW-based nanobarcodes at the readout, because its progression currently substantially lags behind the other merits’ progression.

### 2.1. Encoding and Sensing of Magnetic Nanowire (MNW)-Based Nanobarcodes

Each magnetic nanowire (MNW)-based nanobarcode is made of a collection of MNWs, where the magnetic properties of each MNW and the intra-magnetic interactions can be used for encoding [40,41]. The most favorable magnetic signatures for nanobarcoding are those that can be rapidly measured, with high repeatability, to fit the daily applications as expected for nanobarcodes. This requirement limits the number of magnetic measurements to a few applicable measurements, which can be categorized into the following two groups: (1) DC measurements, and (2) AC measurements. The DC measurements usually need simpler equipment, and they have been widely used for the magnetic characterization of MNWs. As a result, there has been much progress in the development of instruments for fast and repeatable DC measurements. The DC measurements include hysteresis loop measurements, the first-order reversal curve (FORC) method [42,43,44], remanence curve method [45,46], and, most recently, the projection method and the backward remanence method [34,47]. The AC measurements are magnetic particle spectroscopy and ferromagnetic resonance spectroscopy, as the most well-established and common methods that might be transferrable to daily applications [23,48,49].

#### 2.1.1. DC Measurements

**Figure 2:**
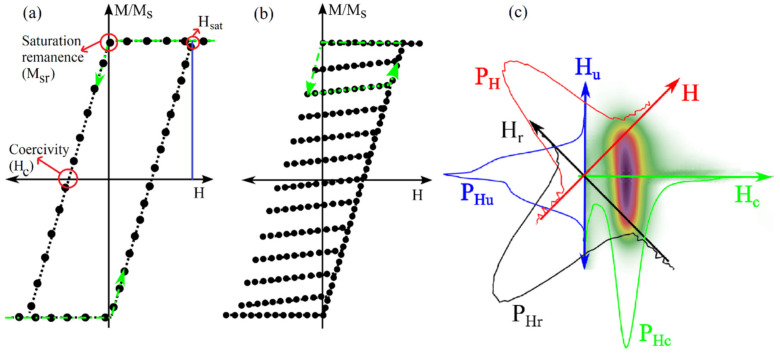
Schematically depicting the hysteresis loop method (**a**) and the FORC method (**b**,**c**), where (**b**) is the FORC data collection and (**c**) is a FORC heat-map. In subfigures (**a**,**b**), the green arrows show the data acquisition direction. In subfigure (**c**), the red distribution is the projection of the FORC heat-map on the applied field (H), the blue distribution is the projection of the FORC heat-map on the interaction field axis (interaction field distribution), the black distribution is the projection of the FORC heat-map on the reversal field (Hr), and the green distribution is the projection of the FORC heat-map on the coercivity axis (coercivity distribution). Figure adapted from [34,35].

**Figure 3:**
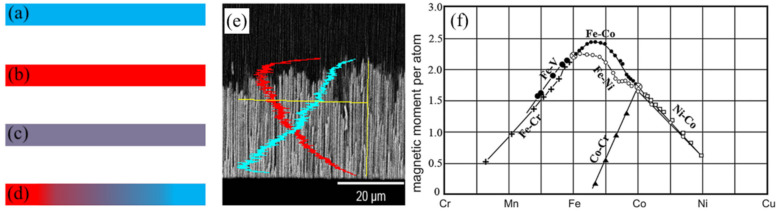
Demonstrating different approaches for tailoring the magnetization saturation and the coercivity of MNWs for encoding. (**a**,**b**) Single component MNWs; (**c**) multi-component or alloyed MNWs; (**d**) MNWs with modulated composition; (**e**) SEM image of modulated composition MNWs, adapted from [74]; and (**f**) represents a Slater–Pauling curve illustrating the dependency of the magnetic moment on the composition, adapted from [58].

The hysteresis loop measurement is the most popular and fastest method for magnetic property extraction, and it provides the saturation magnetization and the coercivity of any sample. Figure 2a schematically illustrates the hysteresis loop method. The saturation magnetization is a function of the MNW composition, as shown in Figure 3a–e. Making MNWs as alloys of one magnetic (such as cobalt or iron) and one non-magnetic component allows the tailoring of the saturation magnetization from zero to the saturation magnetization of the magnetic component [50]. The common magnetic and non-magnetic components that can be easily co-electrodeposited are (1) iron with gold [31,51] or copper [52], (2) nickel with gold [53] or copper [54,55], or (3) cobalt with gold or copper [56]. 

Tailoring the saturation magnetization is best to be conducted using magnetic components with high saturation magnetization, such as iron, if the other components are non-magnetic [57,58,59]. In this case, the saturation magnetization can be tailored over a wider range, from nearly zero up to the saturation magnetization of the magnetic component. Therefore, among all of the compositions, alloys containing iron might be more favorable as they have high saturation magnetization, leading to a wider achievable range of saturation magnetizations. Note that the alloyed MNWs were also made of both magnetic components, such as iron–nickel [60,61], iron–cobalt [62,63], nickel–cobalt [64,65], or iron–cobalt–nickel [66]. When both components are magnetic, the saturation magnetization range will be limited to the minimum and maximum saturation magnetization of the components, except iron–cobalt MNWs with a 2:1 atomic ratio that leads to higher saturation magnetization [63,67,68], as shown in Figure 3f. Generally speaking, having both magnetic components does not provide much flexibility to tailor the saturation magnetization as the encoding parameters. This is also valid when the MNWs are made of three components, such as iron, nickel, and cobalt, as trinary [69,70,71]. However, these cases are very useful to tailor other magnetic properties, such as coercivity, where, as an example, Permalloy (iron–nickel with a 1:4 atomic ratio) is one of the most popular compositions [61,67,72,73].

**Figure 4:**
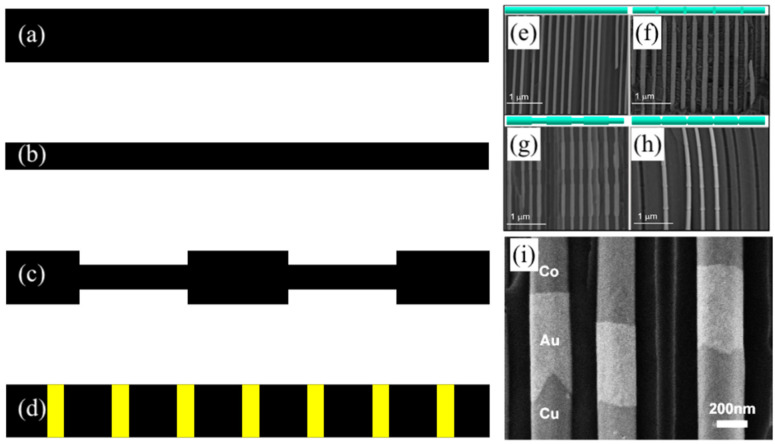
Illustrating the different techniques for tailoring the coercivity of the MNWs, where (**a**,**b**) changing the diameter (or aspect ratio) of single diameter MNWs; (**c**) modulated diameter MNWs; (**d**) multi-segmented MNWs; (**e**–**h**) SEM images of varying and modulating the MNWs diameter, adapted from [95]; and (**i**) a SEM image of multi-segmented MNWs, adapted from [96].

Coercivity is another important magnetic property that determines how resilient the spins are against changing their direction. Simply, coercivity is the required magnetic field to rotate the magnetization 90 degrees, or for very anisotropic samples, it is the field required to produce equal “up” and “down” spins. The simplest way to tailor the coercivity is probably to vary the MNW sizes [75,76,77], as shown in Figure 4a,b. For MNWs with a very large diameter, the spins switch via the nucleation and propagation of a magnetic domain wall, which usually requires lower energies, or equivalently, smaller coercivities. As the MNWs diameter increases, the nucleation and propagation of the magnetic domain walls become easier, which causes the coercivity to decrease [78]. This is because MNWs with large diameters hold multiple magnetic domains, leading to the presence of exchange coupling between the magnetic domains [50]. The exchange coupling contributes to the coercivity that is proportional to the inverse square of the MNWs diameter. Thus, for diameters larger than the critical diameter (the diameter in which the MNWs are a single domain), the coercivity decreases as the diameter increases [79]. Note, once the MNWs diameter becomes smaller than the critical diameter, all spins rotate simultaneously. In this case, which is also known as coherent rotation, the coercivity significantly increases to large values. It should be mentioned that the MNWs length can also impact the reversal mechanism of the spins (i.e. transverse well mode instead of the coherent mode if the length is very long) [80,81]. However, the effects of the length on coercivity are usually taken out, because the definition of MNW obligates a much longer length compared to the diameter. Note, when the length is much longer than the diameter, the shape anisotropy is constant. Thus, the coercivity becomes independent of the length.

Inducing any chemical or physical changes/mismatches that facilitate or hinder the switching direction of the spins would result in tailoring the coercivity [72,82]. An example for the chemical approach is to synthesize MNWs with different compositions or crystal structures [83,84,85,86]. For instance, it was shown that by varying the pH of the electrolyte during electrodeposition, one can manipulate the crystal structure of cobalt MNWs from hexagonal close-packed (hcp), to a mixture of hcp and face-center-cubic (fcc), to a purely fcc crystal structure [85,87]. Over the last few years, the physical approaches for tailoring the coercivity have been intensively studied. The basic for physical approaches is to pin magnetic domain walls by inducing a discontinuity; a few examples are depicted in Figure 4. The examples for physical approaches are diameter modulation (an MNW with multiple diameters along its length) [88,89], multi-segmented [90,91], inducing notches [92,93], and interconnecting MNWs [29,94]. Figure 4 shows some proposed attempts for engineering the MNWs coercivity, by taking benefit of the following: (a–b and e–f) varying diameters, (c and g–h) modulating the diameter, and (d and i) multi-segmenting the MNWs. In all these approaches, the magnetic domain walls are being pinned at the transition sites, which generally leads to an increase in coercivity. Thus, it would be interesting to combine the aforementioned approaches for tailoring the coercivity over a much wider range than the current state-of-the-art.

The first-order reversal curve (FORC) method is another DC measurement that has been broadly used for the qualitative, and partially quantitative, description of magnetic signatures [97,98,99], as shown in Figure 2b,c. In the context of the FORC method, the hysteresis loop of a magnetic nanobarcode can be considered as a picture, where each pixel is the contribution of a hysteron, such as an individual MNW, which builds the whole hysteresis loop area. Experimentally, the FORC method scans the whole area of the hysteresis loop in a two-dimensional fashion, using the following two magnetic fields: (1) reversal field, and (2) applied field [100]. Once the magnetic responses are measured, in terms of the reversal and applied fields, the second derivative of the magnetic responses are taken [101], and the results are plotted as heat-maps versus the reversal field and the applied field.

When plotting each pixel (i.e., a single MNW switching), in terms of the reversal and applied field, the reversal field is the field in which magnetization switches from +1 to −1, while the applied field is the field in which the magnetization switches reversely. In other words, half of their difference (equivalently, the width) is the coercivity, and half of their summation (equivalently, the horizontal shift) is the interaction field [42,100,102]. According to these definitions of the coercivity and interaction fields, the FORC heat-maps can be plotted in the coercivity interaction field plane, which is a 45-degree rotation of the reversal-applied fields plane. Conceptually, the FORC heat-maps indeed determine the probability of finding an MNW with a specific coercivity and interaction field pair. Consequently, since there are many MNWs with different coercivities and under different interaction fields, the coercivity and interaction fields are represented as distributions that have been used as magnetic signatures [103,104,105].

As mentioned earlier, the FORC heat-maps have been broadly used as qualitative descriptions of MNWs magnetic signatures. For quantitative description, the heat-maps are projected onto the coercivity and interaction fields in order to calculate the coercivity and interaction field distributions, and to be used as quantitative signatures [106,107]. This detailed analysis of the FORC method has been known as a very powerful probe for analyzing the magnetic signatures of many complex MNW-based nanobarcodes. As a result, the FORC method became a very promising method for the reliable sensing of nanobarcodes. The major advantage of the FORC method is that it provides the magnetic signatures (coercivity and interaction field) as distributions rather than single values. It is very useful because the measured signatures can be decoded [9]. This detailed sensing of magnetic nanobarcodes, provided by the FORC method, is accompanied with unpleasant downsides that dramatically hinder the usability of the FORC method for practical applications [41,108]. A few examples of these drawbacks are (1) its slow signature extraction, which makes it extremely inefficient for daily usages, and (2) complex data analysis, which causes artifacts. In this direction, several researches have been conducted, to speed up both the data collection [34,35,109] and data processing [110,111] of the FORC method, which is still a long way to go.

**Figure 5:**
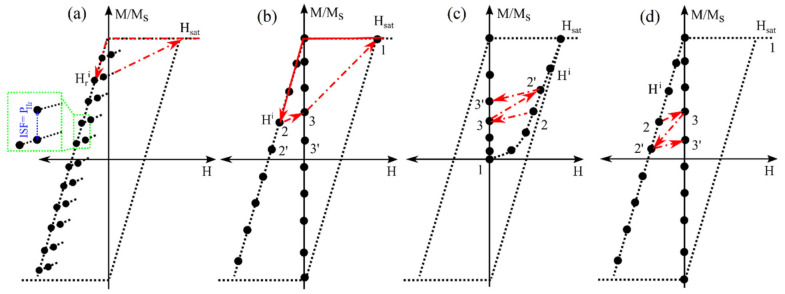
Schematically demonstrating the data collection protocols for (**a**) the projection method, (**b**) backward remanence method, (**c**) isothermal remanence method, and (**d**) DC demagnetization method. The projection method (**a**) provides the irreversible switching (equivalent to the residual magnetization) at the reversal field, Hr. While, the remanence methods (**b**–**d**) provide the residual magnetization at the zero applied field, H, such as points 3 and 3’. The key feature that separates the backward remanence (**b**) from the isothermal remanence (**c**) and the DC demagnetization remanence (**d**) is the saturating the whole system before applying the Hr and removing it.

Among all of the approaches to speed up the FORC measurements, the projection method was proposed, to significantly accelerate the sensing of magnetic nanobarcodes signatures, particularly for biolabeling and nanobarcoding [8,27,34]. A schematic for the projection method is given in Figure 5a. The projection method employs the fundamentals of the FORC method, to extract the irreversible switching field distribution at the reversal field in lieu of coercivity and interaction field distributions. The irreversible switching field distribution as a magnetic signature was found to provide several advantages that are compatible with the expectations for novel nanobarcodes. First, using the projection method, the irreversible switching field distribution can be measured by scanning only the vicinity of the upper branch hysteresis loop—leading to a significant time reduction in comparison to scanning the whole area of the hysteresis loop, as is needed for the FORC method. It was shown that up to five data points along each reversal curve are sufficient to reliably measure the irreversible switching field distribution, which leads to a factor of 50–100X faster measurements compared to the FORC method [34]. Second, the projection method requires only one derivative to calculate the irreversible switching field distribution, while the FORC method requires two sequential derivatives followed by an integral. Due to the measurement noise, the FORC signatures are accompanied by artifacts, which are still elusive [35]. Last, but not least, the projection method indeed measures both the reversible and irreversible switching field distributions, and decomposes them. Since the irreversible switching field is residual magnetization, it is exclusively generated by the MNWs in the barcode, while the reversible switching field could be the superposition of the MNWs reversible response and the surrounding materials. Thus, the projection method provides a better signal-to-noise ratio that is suitable for further miniaturizing the nanobarcode sizes by excluding the background signals. Note, the irreversible switching field distribution is a function of the MNWs coercivity and the interaction fields between them. Therefore, in addition to the aforementioned parameters for tailoring the coercivity, the irreversible switching field distribution can be further tailored by tuning the interaction fields (e.g., by varying the interwire distance) within the MNWs, leading to a more expandable encoding capability [35,112,113].

It should be emphasized that the projection method measures the irreversible switching field distribution at the reversal field. The advantages of the irreversible switching field distribution for nanobarcoding have attracted attention towards measuring the irreversible switching field distribution at zero field, which is also known as the backward remanence measurement [5,6], as shown in Figure 5b. Even though the backward remanence method measures the remanence (magnetization at zero field), it is different from other remanence methods, such as isothermal remanence [114,115] (Figure 5c) or DC demagnetization remanence [116,117] (Figure 5d). Indeed, the backward remanence measurement measures the remanence in a more restricted method that was shown to be more reproducible [34], which is necessary for reliable sensing of magnetic nanobarcodes. The only difference between the backward remanence measurement and the other remanence method is that it saturates the magnetic nanobarcode at each step before applying and removing the field [6]; Figure 5b–d schematically shows the data collections for each of these remanence methods for comparison. This suppresses the stochastic effects of MNWs magnetization in an array, leading to more reliable sensing [5,6].

#### 2.1.2. AC Measurements

**Figure 6:**
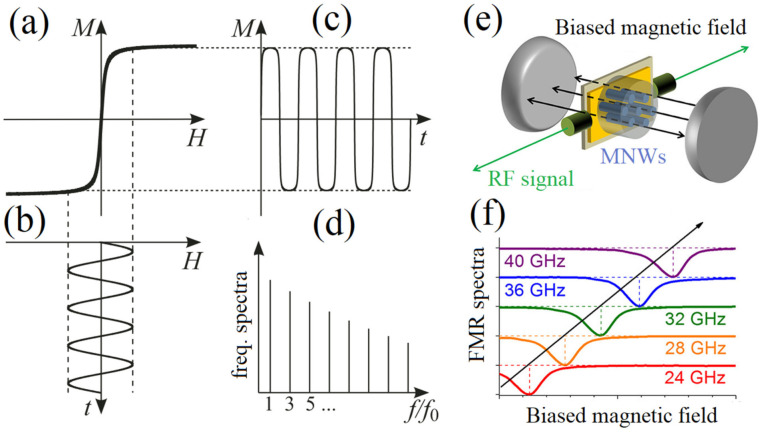
Schematically demonstrating (**a**–**d**) magnetic particle spectroscopy data, adapted from [127], and (**e**,**f**) ferromagnetic resonance spectroscopy data, adapted from [123]. In magnetic particle spectroscopy, superparamagnetic nanoparticles are exposed to an alternating magnetic field (**b**), which forces them to oscillate (**c**). By linearizing their response in frequency domain, multiple peaks appear at odd higher frequencies (**d**) that are being used for sensing them. In magnetic resonance spectroscopy, the MNWs are exposed to an RF signal while a biased magnetic field is applied. By sweeping the RF signal frequency or the biased magnetic field magnitude, the RF absorption of the MNWs varies due to their spins’ precession, where the absorption signal is being used for sensing the MNWs.

AC measurements apply an alternating magnetic field and measure the response of magnetic nanobarcodes at different frequencies or a biased magnetic field. The two widely used AC measurements are magnetic particles spectroscopy [118,119,120], in Figure 6a–d, and magnetic resonance spectroscopy [121,122,123], in Figure 6e,f. Magnetic particles spectroscopy applies an alternating magnetic field, using a magnetic coil at a single frequency (sometimes two frequencies [124,125]), shown in Figure 6c, and measures the magnetic responses in real time, in Figure 6b, and the frequency domain, as shown in Figure 6d. For superparamagnetic nanoparticles, where the coercivity is zero, as in Figure 6a, the magnetization will be a function of odd multiplication of the applied frequency, which aims to sense the magnetic response of the magnetic nanoparticles and distinguish it from the applied signal [126]. Sensing the MNWs using magnetic particles spectroscopy is practically very challenging; this is because the non-zero coercivity of the MNWs causes a nonlinear dynamic response that cannot reliably be sensed and distinguished from the applied field. Furthermore, the MNWs have a non-zero coercivity, from a hundred Oe to several hundred Oe, which mandates a very large AC field for AC oscillations. Applying a large AC field at a high frequency causes heat generation, due to eddy currents. To avoid the eddy currents and to be able to use the magnetic particles spectroscopy for nanobarcoding, the MNWs must have small coercivity. However, this limits the range of encoding, leading to a limited magnetic nanobarcode that is not favorable for nanobarcoding applications.

Another AC measurement for sensing magnetic nanobarcodes is magnetic resonance spectroscopy [123,128], also known as ferromagnetic resonance measurement, shown in Figure 6e,f. Magnetic resonance spectroscopy is developed based on the traditional radiofrequency (RF) identification method, which uses the AC magnetic field of a radio-frequency signal, in the either presence or absence of a DC magnetic field, to sense the magnetic nanobarcodes. By varying the DC magnetic field, the resonance frequency of the MNWs change, and that can be used as an extra degree of freedom for secure sensing [129]. Magnetic resonance spectroscopy could be faster compared to the DC measurements for sensing. However, it tends to have a poor signal-to-noise ratio and short distance sensing, due to absorption/attenuation of the RF signals. Indeed, since magnetic resonance spectroscopy uses an RF signal for the stimulation, it inherently has the limitations of traditional RF identification.

### 2.2. Decoding of Magnetic Nanobarcodes

As discussed in the previous section, there is a trade-off between fast sensing and reliable sensing. A solution for avoiding this trade-off is to reliably sense multiple magnetic nanobarcodes, to speed up the decoding by reducing the number of required readouts/measurements. In contrast to the huge progress in the encoding and sensing of magnetic nanobarcodes, the reliable decoding of them has not received much attention, even though it is crucial for the commercial transition [5,6]. Furthermore, establishing a roadmap for the reliable decoding of multiple nanobarcodes is not only beneficial for the magnetic nanobarcodes, but other nanobarcodes can also take benefits from this, to ramp up the authentication speed. Here, we again discuss the recent works to focus on the MNW-based nanobarcodes.

For reliable decoding of multiple unknown MNW-based nanobarcodes, it is necessary for the magnetic nanobarcodes to have distinct features, with minimum overlapping. This requirement discards the hysteresis loop measurement as it only provides single values, for example, saturation magnetization or coercivity. Aside from the fact that the saturation magnetization and coercivity are well known and have strong magnetic signatures for encoding, they are insufficient for reliable decoding, especially when there is more than one nanobarcode at the readout [5,6,7]. For example, assume the scanner reads the saturation magnetization 100 emu/cc. Since it is a single value, it does not indicate if there was only one nanobarcode with 100 emu/cc or two nanobarcodes with 50 emu/cc for each, and so on. This drawback restricts the application of the hysteresis loop measurement, regardless of it being a fast, easy, and cheap sensing method. As a result, the sensing methods, such as the projection method, that provide distributions stand out. Practically, the most favorable distributions are those that can be tailored using multiple parameters, such as saturation magnetization, coercivity, and interaction fields, to provide higher flexibilities for encoding.

**Figure 7:**
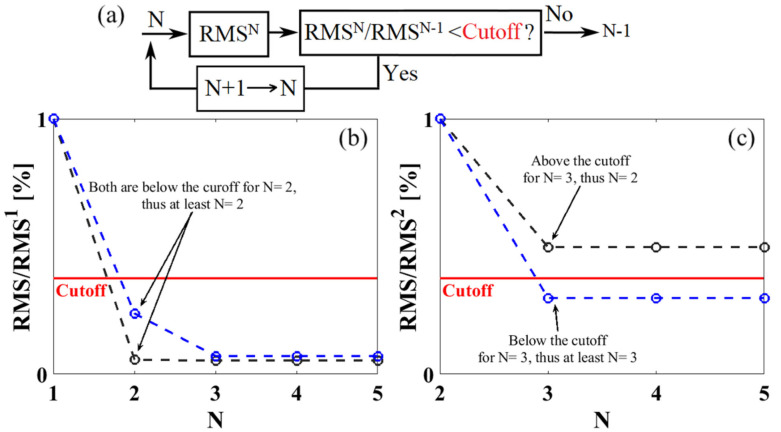
Depicting a decoding method based on using the fitting quality (RMS) as an indicator for determining the number of nanobarcodes at the readout, (**a**) a flowchart for decoding and (**b**,**c**) data analysis for finding the number of the nanobarcodes were produced. The algorithm assumes that one (N = 1) nanobarcode exists, thus, it fits the data with one Gaussian function and calculates the RMS1. Then, it increases N to two, and it calculates RMS2. If the RMS2/RMS1 (**b**) is larger than the cutoff value, there was only one nanobarcode at the readout. Otherwise, there are at least two nanobarcodes and the procedure must be repeated for N=3, which means RMS3/RMS2 must be evaluated (**c**).

The key for the reliable decoding of multiple nanobarcodes is that the readout signal of a combination must be a linear superposition of the individual components. This is usually achievable because the interwire distance between the MNWs (usually in order of 500 nm) in a nanobarcode is several orders of magnitude smaller than the distance between the nanobarcodes (it is at least the thickness of the nanobarcode, which could be in order of 1 mm). This allows the readout signal to be a linear superposition of the signatures of the composed nanobarcodes/subdivisions. Indeed, the challenge is to determine the number of nanobarcodes; Figure 7a shows a simple flowchart for this purpose. This is because increasing the number of nanobarcodes improves the fitting quality, leading to unbounded values for the number of nanobarcodes. To overcome this challenge, it was proposed to use the degree of the fitting quality (i.e., the root mean square, RMS) improvement as an indicator of the likelihood for having the expected number of nanobarcodes [130,131,132], as shown in Figure 7b,c. In other words, it is true that the fitting quality improves as the number of nanobarcodes increases (under-fitting); however, this improvement will not be significant as the fit number surpasses the actual number of codes (over-fitting). To overcome this, one of the proposed techniques is to consider a cutoff value for the improvement in the RMS [5,6]. Therefore, by selecting a cutoff for the RMS, to differentiate between the under-fitting and over-fitting, one can predict the number of the nanobarcodes at the readout; Figure 7 schematically illustrates such algorithms for a readout signal of two nanobarcodes.

Figure 7 schematically illustrates the procedure for decoding using a cutoff value. One first assumes that there is only one nanobarcode (N = 1) at the readout, and the measured remanence spectrum is fit to one Gaussian function to find the fitting parameters by optimizing RMS^1^, where superscript one indicates N = 1. Next, N is increased to 2 and the new optimum RMS error, RMS^2^, is calculated. Then, RMS^2^ is compared with RMS^1^ to determine how much the RMS error decreased, by increasing N from 1 to 2. If the reduction meets the cutoff, then there are at least two nanobarcodes at the readout (N ≥ 2), as shown in Figure 7b. Then, it is necessary to increase N to 3 and repeat the same procedure, to determine whether or not there are more nanobarcodes present. Note, at this step, RMS^3^ and RMS^2^ must be considered, and their ratio must be compared with the cutoff value, as in Figure 6c. If the reduction in RMS^3^ compared to RMS^2^ was not sufficient, the decoding process can be terminated, because it would appear that only two nanobarcodes were present at the readout (N = 2). This process must be continued until the ratio of RMS^N^-to-RMS^N-1^ is no longer smaller than the cutoff value. The main drawback of this technique for reliable decoding is finding the correct value for the cutoff. For example, as the number of nanobarcodes at the readout increases, the magnetic signatures start overlapping, which makes the decoding difficult. It should be emphasized that this drawback is not limited only to magnetic nanobarcodes, as the reliable decoding of any nanobarcodes suffers from this problem. To resolve this problem, we proposed two alternatives. The first alternative is to use a floating cutoff value, which is a function of the predicted number of nanobarcodes. The second alternative, which could be a more effective approach, is to eliminate the need for a cutoff, which could be accomplished by using the artificial intelligent (AI) or the machine learning (ML) approaches. To accelerate the transition of MNW-based nanobarcodes to real-life applications, the reliable decoding of multiple nanobarcodes demands a huge amount of attention, with many research opportunities in computer science and signal processing domains, which are expected to flourish soon.

## 3. Summary and Outlook

Though considerable progress and advances have been recently made toward the encoding/sensing/decoding of MNW-based nanobarcodes, there are still challenges for their use in nanobarcoding applications that need to be addressed to achieve practical translation. The extremely important merits of promising nanobarcodes are as follows: (1) expandable encoding capability, (2) fast sensing with minimal background noises, and (3) reliable decoding of multiple nanobarcodes simultaneously present at the scanner. The encoding capability and sensing merits are inherently correlated, as the former is the targeted magnetic property/signature and the latter is the magnetic measurement method for sensing/measuring the targeted property. The main considerations that must be taken into account when choosing the proper property/signature and sensing/measurement are the stability over time, expandability, and the speed and reproducibility of the signature, in addition to the cost, ease of use, and portability of the sensing instruments. These criteria make many magnetic signatures unusable, and leave a few options available, which can be categorized based on the type of sensing methods, which are DC and AC methods. Basically, the DC sensing methods are more reliable and simpler, but slower than the AC sensing methods. More importantly, the DC methods intuitively provide sensing of the hidden MNW-based nanobarcodes, with higher signal-to-noise ratios compared to the AC methods.

The need for faster sensing, based on DC methods, that provides distributions, instead of single values, to achieve reliable decoding, eliminates the hysteresis loop and first-order reversal curve (FORC) methods, which are brief and slow, respectively. Among all, the projection method and backward remanence deem more promising, as they are as fast as the hysteresis loop method, companied by detailed analyses similar to the FORC method. To further enhance the rapid authentication, it is essential to be able to reliably decode the readout signals from multiple nanobarcodes simultaneously present at the scanner. This requirement demands other fields, such as signal processing from electrical engineering and machine learning or artificial intelligence from computer science, into magnetism, to facilitate the realization of MNW-nanobarcodes translation to daily applications. Indeed, despite the huge progress in the encoding and sensing of MNW-based nanobarcodes, the reliable decoding of multiple MNW-based nanobarcodes is still in its rudimentary stage and requires much exploration.

## Data Availability

Not applicable.
